# Assessing the Mental Impact and Burnout among Physicians during the COVID-19 Pandemic: A Developing Country Single-Center Experience

**DOI:** 10.4269/ajtmh.21-0141

**Published:** 2021-04-22

**Authors:** Muhammad Sohaib Asghar, Farah Yasmin, Haris Alvi, Syed Muhammad Ismail Shah, Kashish Malhotra, Syed Ali Farhan, Syed Anosh Ali Naqvi, Rabail Yaseen, Saira Anwar, Uzma Rasheed

**Affiliations:** 1 Department of Internal Medicine, Dow University of Health Sciences, Karachi, Pakistan;; 2 Department of Internal Medicine, Ziauddin Medical University, Karachi, Pakistan;; 3 Department of Internal Medicine, Dayanand Medical College, Ludhiana, India;; 4 Dow International Medical College, Dow University of Health Sciences, Karachi, Pakistan;; 5 Department of Internal Medicine, Liaquat National Hospital & Medical College, Karachi, Pakistan

## Abstract

Health-care workers are on the front line to combat the peculiar coronavirus disease-19 (COVID-19) pandemic and are susceptible to acquiring this infection. This study is aimed at documenting the effect of “coronaphobia” on mental well-being and to report burnout among physicians. The study was conducted as a cross-sectional survey between November 17, 2020 and January 1, 2021 via a Google form distributed among the physicians of a tertiary care hospital, in Karachi, Pakistan. The Warwick-Edinburgh Mental Well-being Scale (WEMWBS) was used to assess the mental well-being of physicians. Burnout was documented by using the Maslach Burnout Inventory Human Services Survey for Medical Personnel. Eighty-seven physicians participated in the survey (mean age, 30.9 ± 7.3 years). The mean WEMWBS score of the study participants was 51.6 ± 10.8. Regarding the WEMWBS, emotional exhaustion was observed in 54% (*N* = 47) of participants, depersonalization in 77% (*N* = 67), and low personal accomplishment was reported in 31% (*N* = 27) of participants. The results of the survey further highlight that depersonalization, emotional exhaustion, and low personal accomplishment were associated significantly with a history of COVID-19 infection and COVID-19 postings. Hence, immediate measures are required to reduce the burnout among physicians while battling the second wave of the pandemic.

## INTRODUCTION

The novel coronavirus disease 2019 (COVID-19) pandemic, elicited by severe acute respiratory syndrome coronavirus 2 (SARS-COV-2), has become a worldwide burden.^1^ The virus has transmitted rapidly through the entire globe, affecting more than 202 countries.^2^ Health-care workers are on the front line to combat this virus and they have a high chance of acquiring the infection while taking care of affected patients or coming in direct contact with their biological specimens.^3^ The psychological response of health-care workers to a global pandemic is dependent on numerous factors, such as acquiring the virus, the probability of transmitting it to their loved ones, lack or scarcity of personal protective equipment, excruciatingly long working hours, deficiency of diagnostic testing among health-care workers, and paucity of a definitive cure, leading to the development of fear, anxiety, hysteria, depression, and post-traumatic stress disorder (PTSD) among health-care workers.^1,4–6^ A similar study was conducted in China among a characteristic number of health-care workers reporting depression (50.4%), anxiety (44.6%), insomnia (34%), and discomfort (71.5%).^7^ All these mental ailments not only influence the working ability of health-care individuals, but also their attention span, decision-making ability, and understanding, and thus also affecting the mental, physical, and psychological well-being of workers.^4,8^

“Coronaphobia,” although a non-medical term, has newly evolved to denote the psychological impact of this pandemic.^9^ It is defined as an excessive triggered response of fear of contracting the virus, leading to physiological, cognitive, and behavioral changes causing significant stress, safety-seeking behaviors, avoidance of public places, and marked impairment in daily life functioning.^9,10^

In 2003, about 18% to 57% of health-care individuals during the outbreak of SARS underwent severe emotional and psychiatric trauma that affected their mental health.^6^ A study conducted by Carmassi et al.^11^ regarding PTSD among health-care workers during the COVID pandemic concluded the presence of PTSD, particularly among nursing staff, who have prolonged exposure to patients, less work experience, female gender, single marital status, and are quarantined or shunned in their neighborhoods because of their hospital work. In the early days of the COVID-19 pandemic, affected health-care workers accounted for 29% of all hospitalized individuals.^4^ All the factors discussed earlier are known to be associated with mental well-being and burnout among physicians. This study aimed at documenting the effect of coronaphobia on mental well-being and reporting the burnout among the physicians of a tertiary care hospital with the largest COVID-19 isolation unit in Karachi, Pakistan. A secondary aim was to evaluate the association between sociodemographic factors with burnout and coronaphobia among health-care physicians.

## MATERIALS AND METHODS

This study was conducted as a cross-sectional survey of physicians of a tertiary care hospital in Karachi, Pakistan, between November 17, 2020 and January 1, 2021 during the second wave of the pandemic in this region. The sample size of 80 was calculated by using the Rao soft digital sample size calculator,^12^ with a margin of error of 5%, a 95% CI, a response distribution of 50%, and a population size of 100. All physicians of either gender (i.e., male or female) were included in the study, whereas those who refused to give informed consent were excluded. The non-probability convenient-based sample technique was used to collect data. A questionnaire was developed through an online platform via Google forms and was e-mailed to all physicians working in the hospital. A pilot survey was also conducted on 16 physicians, and any necessary modifications were made throughout its course. The finalized questionnaire was concise, easily understandable, and took 3 to 5 minutes to complete. The questionnaire comprised a brief introductory paragraph stating the aims of the study, declaration of anonymity and confidentiality, and a mandatory informed consent followed by the items of the survey.

The questionnaire consisted of two scales to assess mental well-being and document the burnout among the participants. The Warwick–Edinburgh Mental Well-being Scale (WEMWBS) was used to assess the impact of coronaphobia on physicians’ mental well-being.^13^ It consists of 14 questions (on a scale of 1–5), with a cumulative score of 70. The score is validated and reliable according to many previous studies (Cronbach’s alpha, 0.87; re-test reliability, 0.83).^13,14^ A score of more than 50 is considered good mental health, whereas a score less than 43 signifies poor mental health. Burnout was documented by using the Maslach Burnout Inventory Human Services Survey for Medical Personnel (MBI-HSS MP). There were 22 questions that were categorized as emotional exhaustion (nine questions), depersonalization (five questions), and personal accomplishment (eight questions). Each question had seven responses (scale, 0–6). The maximum cumulative scores were 54, 30, and 48, respectively, for all the three categories. After recording the scores, they were graded further into low, moderate, and high according to the cutoffs determined by the manual.^15^ The manual is validated for use as a gold standard in reporting burnout among medical personnel, with a Cronbach’s alpha of 0.86.^16,17^ The cutoff set for emotional exhaustion was high if more than 27, moderate when between 17 and 26, and low if less than 17. Depersonalization was also set as high (> 13), moderate (7–12), and low (< 7). The grading of personal accomplishment was high if more than 39, moderate when between 32 and 38, and low if less than 32. Greater emotional exhaustion and depersonalization contributed to burnout, whereas greater personal accomplishment reduced burnout. The responses of the study participants were gathered and compared among the effect modifiers. All analyses were conducted using the Statistical Package for Social Science v. 25.0 (IBM Corp., Armonk, NY). An independent sample *t*-test was used to estimate quantitative measures among two variables whereas one-way analysis of variance was used for three variables. Fisher’s exact test and the χ^2^ test were used for qualitative measures. Spearman’s correlation was applied to link mental well-being scores with the grading of burnout. Multiple linear regression was applied to determine an association between study variables and mental well-being scores. *P* < 0.05 was considered statistically significant.

## RESULTS

Eighty-seven physicians participated in the survey (mean age, 30.87 ± 7.34 years). Of them, 54% (*N* = 47) were female. Most (49%, *N* = 43) were residents, more than half (60%, *N* = 52) of the participants had COVID-19 postings, and one fourth (25%, *N* = 22) was infected with the virus (Table 1). The mean WEMWBS score of the study participants was 51.63 ± 10.79, with significantly higher scores among consultants (*P* = 0.009) and married physicians (*P* = 0.001), and significantly lower scores among those posted to COVID-19 units (*P* = 0.006) or infected with COVID-19 (*P* < 0.001) (Table 2).

**Table 1:** Descriptive statistics of the study participants (*N* = 87)

Effect modifier	*N*	%
Gender		
Male	40	46.0
Female	47	54.0
Designation		
Consultant	18	20.7
Resident	43	49.4
Intern	26	29.9
Posted in COVID units		
Yes	52	59.8
No	35	40.2
Been infected with COVID		
Yes	22	25.3
No	65	74.7
Relationship status		
Single	42	48.3
Married	45	51.7

COVID = coronavirus disease. Mean age of study participants, 30.87 ± 7.34 years.

**Table 2:** Warwick–Edinburgh Mental Well-being Scale scores of the study participants with respect to the effect modifiers

Effect modifier	Mean score ± SD	*P* value
Gender	51.63 ± 10.79	
Male	49.17 ± 11.75	0.054*
Female	53.72 ± 9.53	
Designation		
Consultant	60.05 ± 3.68	0.009†
Resident	48.44 ± 10.27	
Intern	51.07 ± 12.04	
COVID-19 unit postings		
Yes	49.07 ± 12.18	0.006*
No	55.42 ± 6.87	
History of COVID-19 infection		
Yes	38.54 ± 8.37	0.001*
No	56.06 ± 7.39	
Relationship status		
Single	47.83 ± 10.76	0.001*
Married	55.17 ± 9.64	

COVID-19 = coronavirus disease 19.

* Independent sample *t*-test used to compute the *P* value.

† One-way analysis of variance to compute the *P* value.

**Figure 1.**
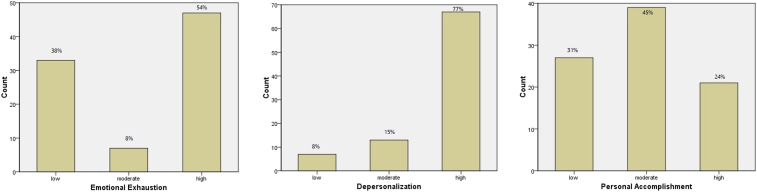
Categorization of Maslach Burnout Inventory Human Services Survey for Medical Personnel scores among the study population (*N* = 87). This figure appears in color at www.ajtmh.org.

Regarding the MBI-HSS MP scale, the mean scores of the participants for emotional exhaustion, depersonalization, and low personal accomplishment were 25.29 ± 12.51, 16.13 ± 5.63, and 33.40 ± 7.03, respectively. Overall, 54% of the participants (*N* = 47) were observed to suffer from high emotional exhaustion, high depersonalization was noted in 77% (*N* = 67), and personal accomplishment was found to be low in 31% (*N* = 27) (Figure 1). Emotional exhaustion was observed to be much greater in residents (*P* = 0.002), in those who worked in COVID-19 units (*P* < 0.001), in those infected with COVID-19 (*P* < 0.001), and in those who were unmarried (*P* = 0.026). Depersonalization was reported more in residents (*P* < 0.001), in those who worked in COVID-19 units (*P* < 0.001), and in those infected with COVID-19 (*P* < 0.001) regardless of gender (*P* = 0.691) or relationship status (*P* = 0.798). Personal accomplishment was observed to be lower in both interns and residents (*P* = 0.015), in those posted in COVID-19 units (*P* = 0.016), in those infected with COVID-19 (*P* < 0.001), and in those who were unmarried (*P* = 0.005) (Table 3, Supplemental Figure 1).

**Table 3:** Maslach Burnout Inventory Human Services Survey for Medical Personnel scores of the study participants with respect to the effect modifiers

Effect modifier	Mean score ± SD	*P* value
Emotional exhaustion	25.29 ± 12.51	–
Male	25.52 ± 11.89	0.877*
Female	25.10 ± 13.14	
Consultant	25.29 ± 12.51	0.002†
Resident	33.23 ± 9.78	
Intern	11.44 ± 3.97	
Worked in COVID unit	30.90 ± 10.11	< 0.001*
Not worked in COVID unit	16.97 ± 11.09	
Infected with COVID	34.27 ± 8.41	< 0.001*
Not infected with COVID	22.26 ± 12.25	
Single	28.38 ± 11.32	0.026*
Married	22.42 ± 12.99	
Depersonalization	16.13 ± 5.63	–
Male	16.40 ± 5.84	0.691*
Female	15.91 ± 5.50	
Consultant	15.66 ± 1.64	< 0.001†
Resident	18.93 ± 4.87	
Intern	11.84 ± 5.89	
Worked in COVID unit	19.07 ± 3.21	< 0.001*
Not worked in COVID unit	11.77 ± 5.64	
Infected with COVID	20.90 ± 3.27	< 0.001*
Not infected with COVID	14.52 ± 5.35	
Single	15.97 ± 6.26	0.798*
Married	16.28 ± 5.03	
Personal accomplishment	33.40 ± 7.03	–
Male	31.80 ± 7.91	0.055*
Female	34.76 ± 5.92	
Consultant	37.61 ± 3.27	0.015†
Resident	32.18 ± 6.23	
Intern	32.50 ± 9.02	
Worked in COVID unit	31.88 ± 6.48	0.016*
Not worked in COVID unit	35.65 ± 7.29	
Infected with COVID	25.09 ± 4.43	< 0.001*
Not infected with COVID	36.21 ± 5.30	
Single	31.21 ± 7.87	0.005*
Married	35.44 ± 5.47	

COVID = coronavirus disease.

* Independent sample *t*-test used to compute the *P* value

† One-way analysis of variance to compute the *P* value.

An interesting finding of our study was the correlation of mental well-being score with burnout score. Emotional exhaustion (*P* < 0.001) and depersonalization (*P* < 0.005) were greater in those with lower mental well-being scores, with personal accomplishment directly proportional to mental well-being (*P* < 0.001) (Table 4). On multiple linear regression, it was found out that mental well-being was associated significantly with COVID-19 infection and personal accomplishment component of the burnout scale (*P* < 0.001) (Supplemental Table 1).

**Table 4:** Correlation of mean Warwick–Edinburgh Mental Well-being Scale scores with the categorization of the Maslach Burnout Inventory Human Services Survey for Medical Personnel

Determinants	Correlation coefficient for mean WEMWBS scores	*P* value
Emotional exhaustion	–0.671	< 0.001*
Depersonalization	–0.300	0.005*
Personal accomplishment	0.791	< 0.001*

WEMWBS = Warwick–Edinburgh Mental Well-being Scale

* Spearman’s correlation used to compute the *P* value.

## DISCUSSION

The health-care workers who have been battling the COVID-19 pandemic on the front line are at an increased risk for contracting the SARS-CoV-2 infection because of its highly contagious nature.^18^ “Coronaphobia” is a new term used to denote the psychological impact of this pandemic, which can be linked to the fear of uncertainty and new genetic mutations, lack of faith in the current health-care infrastructure and resources available, fear of contracting the virus, fear of spreading the virus to the loved ones, avoidance behaviors, and the overabundance of information that may not be fully accurate. These risk factors ultimately manifest by disturbing the daily routine and mental health of the individual as a result of irrational beliefs, maladaptive behaviors, catastrophic interpretation, and social amplifications of risk.^10^ Some of the reasons reported in published literature for adverse psychological health among health-care workers during the COVID-19 pandemic include the stress over an increased duration of working hours and workload, and anxiety over the lack of awareness regarding the pandemic in the community.^6^ Physicians’ fears regarding COVID-19 may also extend to misdiagnosing patients and COVID-19 complications reported by patients.^19^

In this study, we used the WEMWBS scale to evaluate the impact of coronaphobia on the mental health among health-care physicians working on both COVID-19 and non-COVID-19 wards, and demonstrated that the WEMWBS score was significantly less among those posted in COVID-19 units (*P* = 0.006) and infected with SARS-CoV-2 (*P* < 0.001).

The level of burnout in health-care workers can also be used to assess the psychological pressure of working in a stressful environment, sometimes leading to drug abuse, physical illness, depression, or death.^20^ On evaluating burnout status, more than half the participants were observed to experience high levels of emotional exhaustion (54%) and depersonalization (77%). These findings are worrisome, as a meta-analysis has proved emotional exhaustion to correlate negatively with work attitudes among physicians, including professional satisfaction and organizational commitment, quality, and safety, which comprises the time given to patients and management of patient load.^21^ One cross-sectional study reported an overall prevalence of burnout in 76.9% of HIV/AIDS health-care workers. The study discussed that the majority of the HIV/AIDS health-care workers observed their patients die regardless of the treatment they were given, making them feel powerless and more prone to irritability, lack of tolerance to work, anxiety, and depression.^22^ Frontline health-care workers during the pandemic continue to witness the loss of many lives as a result of COVID-19, and are therefore more likely to develop adverse psychological symptoms and burnout resulting from the tremendous number of COVID-19 cases presenting to the hospital.

In addition, resident physicians were reported to experience greater emotional exhaustion (*P* = 0.002) and depersonalization (*P* < 0.001) than consultant physicians in our study. This could be attributed to the fact that these physicians are in the early phase of their medical careers, with limited clinical knowledge and experience. The management of a large number of COVID-19 patients implies increased time pressures and workload, both of which are associated significantly with burnout among resident physicians. This also implies increased job demands, comprising intellectual, emotional, and home–work demands all related to burnout. In addition, the lower resident-to-specialist ratio is one of the work-related factors associated with burnout. The health-care system relies on the contributions of the resident physicians, who are expected to stay in the hospital overnight, whereas the consultant is often on call from home. Their regular residency schedule is also hampered by menial tasks, such as excessive paperwork. Considering there are a limited number of residents available in any medical center, it is not surprising that the overburdened health-care system would require residents to provide both hospital care and do scutwork (i.e., menial tasks) during the COVID-19 pandemic, which then leads to emotional exhaustion and poor mental well-being.^23^ This is also reflected from their lower personal accomplishment scores compared with consulting physicians (*P* = 0.015). In contrast, interns were the least emotionally exhausted, with the lowest depersonalization scores, and scores in personal accomplishment similar to resident physicians. This can be explained by the fact that interns are under the supervision of consulting and resident physicians, and have less expectations regarding their ability to manage patient load and perform complicated clinical procedures. Therefore, they are given easier tasks, leading to better psychological health. Women also tend to have a greater frequency of burnout than men, which was associated with work–home conflict, according to one study.^24^ However, in our study, gender was not associated significantly with high mean MBI-HSS scores (*P* values of 0.877, 0.691, and 0.055 for emotional exhaustion, depersonalization, and personal accomplishment, respectively).

Our study demonstrated that physicians with lower mental well-being scores reported greater emotional exhaustion and depersonalization, with personal accomplishment directly proportional to mental well-being. This represents the correlation of the MBI-HSS MP with the WEMBWS, which needs to be studied further in larger scale studies. In addition, in our study, emotional exhaustion was seen to be less in physicians who were married (*P* = 0.026). Because less emotional exhaustion correlates with greater WEMWBS scores (*P* < 0.001), the WEMWBS scores were also found to be greater in married physicians (*P* = 0.001). This could be explained by the emotional support offered by the partner in the relationship.

Last, the findings of our study are consistent with another study that showed coronaphobia to be a good predictor of psychopathology and a significant contributor to poor mental health.^25^ Urgent action needs to be taken to assess and promote mental well-being among health-care workers because coronaphobia has been associated with suicidal ideation, extreme hopelessness, negative religious coping, alcohol and drug abuse, and emotional distress.^26^ Recently, a man committed suicide in India because of the fear that he had contracted the virus. Other studies conducted in the United States^27^ and Iran^28^ have also linked coronaphobia with depression, anxiety, and poor mental well-being.

## LIMITATIONS

The study was conducted at a single-center institution with a limited sample size and thus may not reflect the findings of larger populations. Larger multicenter studies need to be conducted to evaluate further the findings of our survey. Because no longitudinal follow-up was performed, we could not determine improvement or worsening of psychological symptoms. More studies are needed to understand the long-term psychological implications among health-care workers. Despite these limitations, our study reports critical preliminary information for studying the mental impact of coronaphobia and burnout among physicians during the COVID-19 pandemic.

## CONCLUSION

Coronaphobia causes various psychological and mental impacts on frontline health-care workers. It was evident during the first wave of COVID-19 that resources should be invested to promote the mental health of these frontline professionals, both in terms of prevention and treatment. As reflected by our results, the frequency of burnout is significantly greater in our health-care community. Therefore, immediate measures were carried out to reduce burnout among physicians while preparing for subsequent waves of this pandemic. Several counseling and psychotherapy-based sessions on stress adaptation were provided to those who needed help. Additional training workshops regarding mental health are planned to improve the coping mechanisms of frontline health-care workers. Large-scale study protocols should also be undertaken not only to report but also to address the psychological disturbances through these interventions.

## Supplemental tables

Supplemental materials
