# Supplementary material for: Assessing the Mental Impact and Burnout among Physicians during the COVID-19 Pandemic: A Developing Country Single-Center Experience
Source: Am J Trop Med Hyg. 2021 Apr 22;104(6):2185–9. doi: 10.4269/ajtmh.21-0141 (PMC8176473; doi:10.4269/ajtmh.21-0141)

Supplemental Table 1: Multiple linear regression of study variables with mental well-being score (WEMWBS) being the dependent variable.						
Model	Unstandardized Coefficients (B)	Standard error	Standardized Coefficients	t-statistic	P-value	95% Confidence Interval for B
(Constant)	-3.454	9.007	-	-0.384	0.702	-21.389 – 14.481
Age	0.321	0.170	0.219	1.885	0.063	-0.018 – 0.660
Gender	1.496	1.129	0.069	1.325	0.189	-0.752 – 3.743
Designation	-0.342	1.847	-0.022	-0.185	0.853	-4.020 – 3.336
Did posting in COVID units	2.624	1.495	0.120	1.756	0.083	-0.352 – 5.601
Been infected with COVID	6.026	1.606	0.244	3.751	<0.001*	2.827 – 9.225
Relationship status	1.436	1.278	0.067	1.124	0.265	-1.109 – 3.981
Emotional Exhaustion	0.072	0.094	0.083	0.760	0.449	-0.116 – 0.259
Depersonalization	-0.263	0.247	-0.137	-1.063	0.291	-0.755 – 0.230
Personal Accomplishment	0.885	0.138	0.577	6.420	<0.001*	0.611 – 1.160
* *indicates significant p-value of less than 0.05*						
Abbreviations:- WEMWBS: Warwick–Edinburgh mental well-being scale; COVID: coronavirus disease.						

Supplemental Figure 1

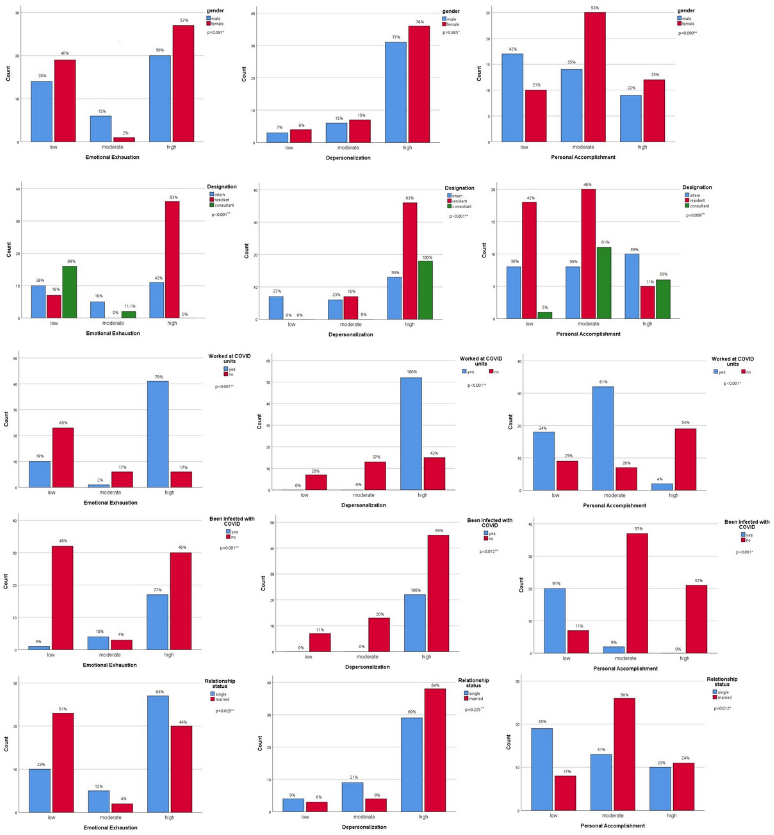

Supplement: Supplementary file 1 [file tpmd210141.SD1.pdf]
